# Costly children: the motivations for parental investment in children in a low fertility context

**DOI:** 10.1186/s41118-020-00111-5

**Published:** 2021-02-17

**Authors:** Anne H. Gauthier, Petra W. de Jong

**Affiliations:** The Netherlands Interdisciplinary Demographic Institute-KNAW and University of Groningen (RUG), Groningen, Netherlands

**Keywords:** Intensive parenting, Qualitative, Parental investment, Low fertility, Cost of children

## Abstract

While the literature has documented a general increase in parental investment in children, both in terms of financial and time investment, the motives for this increase remain unclear. This paper aims at shedding light on these motives by examining parents’ own narratives of their parenting experiences from the vantage point of three theoretical perspectives. In doing so, the paper brings side-by-side the goal of providing children with human and social capital to improve their future labour market prospects, the pressures on parents to conform to new societal standards of good and intensive parenting, and the experience of parenting as part of self-development. The data come from a qualitative study of middle-income parents in Canada and the USA. The results provide some support for each of these perspectives, while also revealing how they jointly help explain parents’ large investment in their children as well as the tensions and contradictions that come with it.

## Introduction

“I will not hide it: everything goes to my children.”

That children have become more costly is no news. Estimates based on time use and expenditures data have shown a major increase in the cost of children for parents in recent decades (Dotti Sani Giulia & Treas, 2016; Gauthier, Smeeding, & Furstenberg, 2004; Kornrich & Furstenberg, 2013). Today’s parents, spend not only more money on their children than in the past, but also devote more time to them (Bianchi, Cohen, Raley, & Nomaguchi, 2004). Moreover, this increase in parental investment into children has been observed in a wide number of different countries and contexts, suggesting the emergence of a new global trend (Cornwell, Gershuny, & Sullivan, 2019).

What is less clear from the literature are the reasons and motives behind this increase in parental investment. For example, while sociologists have been pointing to the emergence of a new social norm of good parenting to explain this trend (Hays, 1996), economists have instead pointed to a preference for children of ‘better quality’ (Becker, 1991). In particular, the needs for providing children with a better education and for better equipping them to confront an increasingly competitive labour market have been suggested to be prime economic motives for this greater parental investment in children (Duncan & Murnane, 2011).

At the same time, and somewhat paradoxically, this increase in parental investment into children has been taking place in the context of increasing individualism, including a growing importance attached to self-growth and self-fulfillment (Lesthaeghe & Meekers, 1987; Mills & Blossfeld, 2013). In other words, what could be expected is for today’s parents to be devoting more time on themselves—rather than more time on their children—in order to fulfill their needs for individual autonomy and self-actualisation (Lesthaeghe, 2010). However, scholars including proponents of the second demographic transition (STD) theory have argued that having children is actually not incompatible with individualistic and post-modernist values (Sobotka, 2008; van de Kaa, 1998). Instead, large parental investment into children, and the experience of parenting as an experience and ‘project’, can be conceived as being part of one’s own personal growth strategy (Wall, 2010). From this perspective, the quest for personal growth can also be posited to be a motive for large parental investment into children.

In short, while the empirical literature provides solid evidence regarding the increase in parental investment into children, the actual explanations remain segmented into distinct theoretical perspectives, each pointing to different drivers and mechanisms. The aim of this paper is to examine the different perspectives and investigate to what extent each of them figures prominently in the discourse of parents as a way of justifying their investment in children. Moreover, by bringing these three perspectives side-by-side, the paper also reveals important interconnections between them. This way, we aim at a more comprehensive understanding of parental investment into children.

To this end, we use data from a large qualitative project aimed at investigating the daily reality of middle-income families in Canada and the USA. The use of qualitative data is particularly appropriate here since it allows us to examine the ‘whys’ and the ‘hows’ of the large time and financial cost of children. In particular, and on the basis of in-depth conversations with mothers, we examine the reasons for their allocation of time and money to their children as well as the ways they reconcile their investment in children with perceived social norms.

We start the paper with a review of the literature on theoretical perspectives on parental investment into children. In particular, we highlight the different driving forces that these perspectives identify to explain parents’ motivations for their allocation of resources to their children. We further link these explanations to other global value changes, especially the rise of individualization and personal responsibility. On the basis of these theoretical considerations, we then move to a description of our data and methods, followed by a presentation of our results. In the last section of the paper, we reflect on the theoretical implications and broader validity of our results.

## Theoretical background

The term parental investment into children refers to the allocation of resources (time and money) by parents to their children, including parents’ management of their children’s risks and opportunities (Bradley & Corwyn, 2004; Gauthier, 2015b). It includes money spent on essential and non-essential goods directly (e.g. clothing) and indirectly for children (e.g. larger utility bill as a result of having a larger family), and it includes time spent doing things with children (e.g. reading with children) and on their behalf (e.g. arranging extra-curricular activities) (Pailhé, Solaz, & Tanturri, 2019). Finally, it includes an important emotional component associated with devoting one’s own attention and energy to children (Cobb-Clark, Salamanca, & Zhu, 2019; Gauthier, 2015b).

As a consequence of its wide nature, parental investment is difficult to conceptualize and tends to be operationalized differently by different disciplines. For example, economists traditionally refer to parental investment in terms of the time and money invested into one’s children (Francesconi & Heckman, 2016).^1^ In contrast, sociologists tend to refer to parental investment in children in terms of the social and cultural capital transmitted from parents to children (Furstenberg, 2005). Psychologists, in turn, tend to refer instead to family process investments, including the resources deployed by parents for the sustenance, stimulation, and socioeconomic support of children (Bradley & Corwyn, 2004).

Regardless of the interpretation of parental investment, all disciplines point to an actual increase in parental investment over the past decades. The key question is therefore: why have parents been devoting an increasing amount of resources to their children? In this paper, we focus on three explanations for this trend.^2^

### Future labour market prospects as a motive for parental investment

The first perspective views parents’ investment as a way of increasing children’s future labour market prospects and future ‘returns’. Theoretically, it assumes parents’ investment in their children—especially in their human capital—to be driven by a desire to secure children’s future (in an altruistic way), to preserve or improve a family’s social status, or to ensure support (returns) at an older age (Behrman, Pollak, & Taubman, 1995). These motives have often been evoked in the social stratification literature by which parents’ investment in their children’s human capital (i.e. their education) can be seen as being an instrument of social class reproduction (Albertini & Radl, 2012), a way to upward social mobility (Skirbekk, 2008), and a strategy related to status anxiety (Zuanna, 2007). In the context of the increasing income inequalities that have been observed in most industrialized countries in recent decades, parental investment in their children’s education has thus been argued to be a way for middle-class parents to preserve the social status of the next generation (Napolitano, Pacholok, & Furstenberg, 2014; Rao, 2018).

This investment into children is also central to economic theory on fertility (Becker, 1969, 1991; Werding, 2014), which suggests a preference for children of high ‘quality’ (i.e. more expensive) as opposed to a larger ‘quantity’ of children. This preference, it has been argued, is a result of ‘quality’ children being a desirable feature in itself (children as consumption goods), or because of the expected returns associated with parents’ higher investment in their children (children as investment goods) (Cochrane, 1975). However, this economic approach to family decision-making has been heavily criticized by some, especially its conceptualization of children as consumption goods (starting with Blake, 1968). Yet, and despite these criticisms, the increasing demand and preferences for human capital in offspring continues to be a dominant explanation of fertility decline (Galor, 2012). More generally, the perceived importance of children’s education by parents has been shown to not only have major implications for parents’ allocation of resources, but to also constitutes a major obstacle to fertility, especially in East Asian societies (Anderson & Kohler, 2013).

Investment in the human capital of one’s child is not the only way of providing children with a good head start in life. Providing children with other skills, especially with social capital, has also been argued to be part of the preservation of social class status (Baizan, Dominguez, & Gonzalez, 2014). The work of Lareau (Lareau, 2002, 2003) on child-rearing logic does not stem from an economic tradition, but nonetheless acknowledges the importance of economic motives to parental investment. In particular, the child-rearing logic of ‘concerted cultivation’, she argues, has been adopted by the middle-class as a way “to make sure that their children are not excluded from any opportunity that might eventually contribute to their advancement” (Lareau, 2003, p. 5). In contrast, the child-rearing logic of ‘natural growth’ has been assumed by Lareau to be more characteristic of the working class, to involve lower parental investment and less structured time for children, and to be a better strategy to prepare them for their future (class-related) labour prospects.

### Social pressures (or societal norms) as a motive for parental investment

While the previous perspective points to individual-level desires and preferences, a distinct body of literature has instead been pointing to societal-level pressures for investing in children. Among these studies, changes in parenting ideology and especially the ideology of intensive parenting have been attracting increasing attention in the literature (Gauthier et al., 2020). In a nutshell, this literature argues that societal-level standards of what constitutes a good parent have changed, and that these standards nowadays involve very large parental investments into children (Nomaguchi & Milkie, 2020).

The term ‘intensive mothering’ was coined by Hays (1996) some two decades ago to capture this new parenting ideology and the related pressures on mothers to invest in their children. In particular, this ideology carries a large expectation regarding parents’ time, money and emotional investment in children, as well as expert-guidance to provide the best for the children. In particular, deploying time and financial resources to stimulate children’s development has become part of what a good parent is expected to do (Faircloth, 2010).

In turn, these new standards of good parenting involve large sacrifices from the parents themselves. In particular, time-use studies have shown how the increase in parental time with children has come at the expense of parents’ own personal time and consumption (Nomaguchi & Milkie, 2003). The adherence to this new social norm has also been argued to carry an element of constant anxiety for doing the right thing and the best for children (Warner, 2005), and a feeling of guilt when failing to withhold this standard (Sutherland, 2010). And while there has been a counter movement warning parents about the dangers of ‘hyper-parenting’ for the parents themselves and for the children (Rosenfeld & Wise, 2001), a growing—mostly qualitative—literature has been documenting how pervasive this new norm of good parenting has become (Ennis, 2014; Faircloth, Hoffman, & Layne, 2013).

In addition to changes in parenting ideologies, the other major societal-level change observed in recent decades has been the rise of neo-liberalism, and with it the rise in the value attached to personal responsibility (Parsons Leigh, Gauthier, Iversen, Luhr, & Napolitano, 2018). While the emerging welfare states of the 1950s placed a large responsibility on governments for its citizens’ well-being, the retrenching welfare states of the past decades have instead shifted the responsibility back to individuals (Gillies, 2008; Wall, 2013). More specifically, a ‘good citizen’ has come to be viewed as one who takes responsibility for his/her children’s success, makes the right choices for them, and listens to expert guidance (Barr et al., 2012; Karlsson, Löfdahl, & Prieto, 2013). The politics of parenting and its emphasis on personal responsibility thus converges with the normative intensive parenting ideology, as both expect large parental investment.

### The quest for self-growth as a motive for parental investment

Finally, the third perspective views parental investment in children as a source of personal fulfillment and self-growth (Arendell, 2000). This argument has been made in the context of the transition to parenthood, where becoming a parent is associated with the acquisition of a social identity (Arendell, 2000; Söderberg, Lundgren, Christensson, & Hildingsson, 2013). But it has also been made with regard to child-rearing, by which negotiating today’s challenges and social expectations related to good parenting contributes to a feeling of accomplishment and self-growth (Nomaguchi & Milkie, 2020). For example, recent studies have concluded that a child-centered approach is associated with higher levels of well-being and happiness (Ashton-James, Kushlev, & Dunn, 2013; Le & Impett, 2019). From this point of view, becoming a parent, and the experience of parenting, can be seen as being part of a personal project (Wall, 2010).

A similar argument has been made as part of the SDT theory. The rise in individualism, it has been argued, is not necessarily incompatible with having children (van de Kaa, 2004). Instead, and since the decision to have a child has become a personal choice, becoming a parent can be part of one’s own personal growth strategy. As explained by Sobotka (2008) in his review of the SDT theory, “Having children ceases to be a normatively-bound decision, and it increasingly serves individual self-fulfillment and private joy” (p. 177). Similarly, van de Kaa (1998) argues that having children “may constitute an important element in their [postmaterialist men and women] perception of wellbeing and self-realization” (p. 32).

### Contribution of the paper

While the increase in parental investment into children has been well documented, our understanding of parents’ motives for doing so remains compartmentalized into specific (mostly disciplinary) perspectives. Through an analysis of parents’ own discourses on their parenting experience, this paper contributes to the literature by bringing side-by-side the three different theoretical perspectives on parenting described above. In doing so, the paper provides a rich understanding of the goals, pressures, and dilemmas faced by parents in allocating their resources to their children, which goes beyond the boundaries of each perspective. What is more, while the theoretical literature seems to suggest that the different motives operate separately, our data illustrate how these motives are intertwined in contemporary parenting experiences and discourses.

### The Canadian and American context

The data for this paper come from a qualitative study of middle-income families carried out in Canada and the USA. These two countries were selected in view of the ongoing large academic and societal debates on the ‘threat’ to the middle-class and especially the perceived inability of middle-class parents to transmit their middle class status to their children (New York Times, 2014; Pew Research Center, 2015). In addition, the increase in neo-liberal ideologies in both countries may have also contributed to a re-definition of the notion of what makes a good parent and a good citizen (Gauthier, 2015b; Parsons Leigh et al., 2018).

From a study design perspective, the choice of Canada and the USA constitutes what can be considered as a study of ‘most similar’ cases (Przeworski & Teune, 1982). Institutionally, they are both considered as being a liberal welfare state (Esping-Andersen, 1990) and as providing a relatively low level of governmental support for families (Thévenon, 2011). Economically, they both display relatively high levels of child poverty and income inequality (UNICEF, 2013, 2016), and in recent years have both experienced large economic pressures on their middle class (Gauthier, 2015b). Culturally, they belong to the same English-speaking cluster^3^ in terms of secular-rational values and self-expression (Inglehart & Welzel, 2019). The countries also share to a large extent the importance of hard work for getting ahead in life as captured by the ‘American’ dream or its Canadian equivalent (Corak, 2019).

The two countries also feature dissimilarities, for example in terms of specific governmental programs (e.g. health care system, maternity and parental leave schemes) (Baird & O'Brien, 2015). The demographics of the two countries further differs, with the USA displaying higher levels of teenage fertility, single parenthood, and divorce as compared to Canada (Barbieri & Ouellette, 2012). Moreover, the total fertility rate in the USA has steadily remained close to replacement level in the past decades, while that of Canada has remained around 1.6 children per woman (Rindfuss, Choe, & Brauner-Otto, 2016).^4^

Despite these differences, the common rise in income inequality and neo-liberal ideology in both countries provided a particularly interesting setting to examine the daily reality of middle income families including their investment in children in a North American context. Obviously, this context is also highly heterogeneous with large regional and race/ethnic differences. The nature of our data however prevented us from examining these variations.

## Data and methods

This paper uses qualitative data from the Families in the Middle (FIM) study, which was designed to understand the daily realities of middle-income families in Canada and the USA.^5^ The study was based on a mixed-methods and multi-site design to collect data on a wide range of topics including participants’ financial situation and experiences as parents. The data were collected between 2007 and 2010.

All participants in the study had a middle-income (defined below) and had at least one child aged 10 to 14 years old living in the household. Participants were recruited through various channels (e.g., cultural and sport organizations, schools, local newspapers, and personal contacts) and from various sites. Specifically, data in Canada were collected from urban centers in four provinces (Alberta, British Columbia, Ontario, Quebec) and in the USA from two states (Pennsylvania and Washington). Because of the qualitative nature of the study, the aim was not to construct nationally representative samples, but instead to diversify the sample in terms of its geographical, institutional, and policy context. Obviously, and as mentioned earlier, this does not capture the large heterogeneity inherent to these two countries, but the strategy was to go beyond a single-site study in order to capture the experiences of a wider group of parents.^6^

Participants were first directed to a short online questionnaire which captured basic demographic data and information about their experience as parent. At the end of the questionnaire, they were asked if they would be interested in participating in the qualitative study. Among the participants who indicated that they would be willing to participate in the qualitative study, only those with a middle-income were re-contacted.

For the purpose of the study, a middle-income was defined as a household income within the range of 75 to 150% of the country’s respective family annual median income before taxes (around 60,000 US dollars in 2009).^7^ This corresponds to an income range of 45,000 to 90,000 US dollars and is consistent with the definition of middle-income used in other recent studies (Gauthier, 2015a). However, and as explained below, for the purpose of this study, this income range was narrowed down to better capture respondents facing similar income constraints.

The qualitative interviews were carried out following a semi-structured interview guide which covered a wide range of topics such as neighborhood, daily routines, children’s schooling, parents’ hopes and worries for their children, health and caregiving, and financial circumstances. All interviews were conducted by local fieldworkers, generally took place in the home of the participant, and lasted on average around 75 min. All names mentioned in the “Findings” sections are pseudonyms. Ethics approval from the institutions of each of the principal investigators was obtained for this study.

### Sample

The overall qualitative sample for the FIM study comprises 156 interviews including families of various size and composition (see Gauthier, 2015a for more information).^8^ From this original sample, we retained only participants whose income matched a narrower definition of middle-income (75–125% of the median, corresponding to 45,000 to 75,000 US dollars) (*N* = 115), and within this selection only those with two or three children (*N* = 83).^9^ The justification for doing so resides in the known impact of income and the number of children on parental time availability and resources. For the purpose of this study, we deemed it important to compare families with similar demographics and constraints.

From the remaining sample, we furthermore excluded six cases for which the father was interviewed alone, as the number of male respondents was too low to investigate them separately. Importantly, and while the questions of the interview guide covered parental investment of both parents, this means that what we capture in the paper are the mothers’ voices. These voices are not confined to their own investment into children, but capture their own views, interpretation, and experiences of parenting. Finally, we excluded two additional cases: one involving a grandmother, and one for which the transcript was missing. This resulted in a sub-sample comprised of 75 middle-income mothers with two or three children residing at home, including 36 Canadian and 39 American mothers.

In terms of demographic characteristics, the mothers in the sample had a mean age of 41 years and had an average of 2.4 children residing at home. The large majority of them were married or cohabiting at the time of the survey (75%), had some post-secondary education (84%), were employed (75%), and homeowner (93%). A large proportion of these women furthermore self-defined as being middle-class (85%). All these statistics were comparable across Canada and the USA.^10^ What these average statistics do not reveal is the large heterogeneity among this middle-income group. For example, the occupations among our participants included teaching assistant, nurse, social worker, research technician, accounting manager, and journalist. Still, what the participants all shared was a middle income. This not only constrained to various degrees their investment in their children, but had also implications on what they could realistically hope for their children’s future.

### Methods of analysis

The interviews were transcribed and analyzed in their original language (French or English) and were coded using the qualitative software NVivo. The analytical approach involved identifying patterns and themes emerging from the data. This led to the development of a first set of general codes agreed upon by the whole team of investigators, and subsequently to the development of a more targeted set of codes related to parental investment. During the coding process, the focus was not on the structure or sequencing of the narration, but rather on the content of the participants’ stories and own parenting experiences (Parsons Leigh et al., 2018; Rubin & Rubin, 2005). The analysis was carried out by locating the segments of the interviews that related to parental investment. These segments were analyzed in the context of the participants’ whole story and were subsequently contrasted and compared to unravel commonalities and deviations in the ways participants expressed their views and experiences. In a final step, the qualitative material was viewed in light of the different theoretical perspectives to investigate to what extent the findings empirically underpinned the main premises of each perspective, as well as the interconnections between them.

## Findings

### Costly children

Parents in our study were undoubtedly aware of the high cost of raising children, and of their high time and money investment into their own children. Julie—a married mother of three from Canada—is not unique in stating: “we do a lot for our children … I will not hide it, everything [money] goes to the children”. Similarly, Emily—a married mother of two, also from Canada—captures well her high investment in children in stating: “We really wanted [our children] to have everything that they—we didn’t want them to want for anything… So we really put that priority above other things.” Parents in our sample did not only spend much money on their children, but also devoted large amount of time to them. They spent time in direct interaction with their children, spent hours driving them back and forth from school and various extra-curricular activities, and also devoted much time on their children’s behalf. For example, parents devoted large amounts of time researching opportunities for their children, interacting with school and other institutions, as well as managing the whole family’s schedule. However, the question remains: why are they willing to invest so much in their children, and/or why do they feel they have to?

### Motivation: education and definition of success

In line with the perspective pointing to parental investment towards their children’s future labour market prospects, the perceived importance of education emerged indeed as a key reason for parents’ allocation of time and money to their children. In particular, most mothers expressed hopes that their children would go to college or university, even though—and as shown in another paper based on the FIM data—their middle-income status often did not allow them to afford the cost of their children’s higher education (Napolitano, Furstenberg, & Pacholok, 2014). In our interviews, getting a further education was seen as providing a foundation in life, and a form of investment in the children’s future. As Megan—a married American mother of two—argues: “I mean, our expectation is college: four years. (…) I think it’s a good foundation to have. (…) Nobody can ever take that away from you”. Grace—a divorced mother of two, also from America—goes in the same direction in stating: “If you don’t go to college, you don’t survive”. Mothers not only stressed the importance of education for their children, but also took direct actions to support them, for example by looking for additional resources when their child had learning difficulty, or by talking to the teachers if they had any concerns. Although these actions required a large time investment, they were perceived as key for their children’s future.

However, what also became clear from the data, is that future labour market success is not the only way for mothers to justify their time and money investment in their children. Other—non-economic—markers of success were predominant when mothers described what they hoped for their children. Shannon—a married American mother with two children—for instance endorsed a definition of success that goes beyond economic considerations when stating: “I think to be a success, you just kind of have to be happy and fulfilled and grounded at the same time”. This alternative definition of success was frequently reflected in the terminology that mothers used to describe their hopes for the future of their child. They wished for their children “to be able to do what they want to do”, “to know who they are”, “to be happy”, “to do something they enjoy” and “to be the best they can be”. In turn, these additional markers of success motivated parents’ investment in their children. As Penny—a married mother of three from Canada—summarizes it: “Success is not defined by your degree, but I would expect that going to school and all that is part of being successful, because it helps in the development of who you are”.

In a similar vein, mothers in our sample justified their high allocation of resources to their children by hoping for a happy life for their children, for the fulfillment of their dreams, and as a means to enable the child’s personal growth. In response to the interviewer’s question of how far the participant expected her children to go in terms of education, Jackie—a married mother of three from America—replied: “I would love for them to go to college, if they choose that. (…) Whatever makes them happy I’m okay with it”. Elizabeth—a married mother of two from Canada—takes a slightly different stance, but also emphasizes the non-economic value of education. As she phrases it: “I’d like for them to go to college. That’s what’s going to make them better in what they want to do”. Nicole—a married Canadian mother of three—goes further in stressing the importance of self-growth for her children: “I would really hate for them to get stuck and decide they can’t do anything better than being something less than they are”. In contrast to a strict economic approach, mothers’ motives for investing in their children thus encompassed a more global definition of success, including their children’s self-fulfillment and happiness.

### Motivation: social pressures, parental responsibility, and norms of good parenting

While the above findings point to personal preferences and individual-level considerations as determinants of parental investment, social norms and social pressures were also omnipresent in the participants’ narratives, especially in their descriptions of the challenges associated with being a parent today. Mothers referred for instance to the large consumer pressures to spend more money on children, be it for specific brand-name clothing or electronic gadgets; pressures to spend more time with their children, including pressures to be there for them; pressures to participate in school events; and pressures to conform to standards of good parenting as portrayed in the media and popular magazines.

These high pressures to invest in children often left mothers torn between bowing to pressures and resisting to them. For example, Ashley—a divorced mother of two from Canada—is critical of the normative pressures to invest in children, but at the same time reckoned that she has no choice but to bow to it. As she phrases it: “There’s so much pressure on our kids and ourselves and then we can, you know we can say to our neighbors how great a swimmer our kid is or, whatever it is you know, so I think that’s where society puts pressure”. In a kind of double whammy, failure to conform to expectations is thus perceived by mothers as having severe personal consequences, not only for the child but also to reflect badly on the parent.

Other mothers took a more pragmatic approach in limiting the number of extra-curricular activities in order to preserve some downtime for their children, to carve out more family time, or even to take a stance against all these pressures. For example, Connie—a married mother of two from Canada—explains:

“I think our society’s been so bombarded with this, this and this and cell phones and TVs and video games and parents are just feeling that they have to give, give, give their kids and I, I think a good parent today backs off of that, like really?”.

Sophie—a divorced mother of two from Canada—is even more vocal in being very critical about all the child-centered parenting practices: “The parents, they are always busy bringing their children everywhere: they are slave to their own children! I do not get over it!” These statements however appeared to be the exceptions to the rule, as most mothers fully endorsed the high parental investment inherent in societal standards of good parenting.

The pressure to conform to social expectations of good parenting often resulted in much anxiety. For example, while reading on best parenting practices and keeping up with expert advice was seen as essential, mothers also expressed a feeling of being overwhelmed by the amount of information. Julia—a divorced mother of three from America—reflects on this in saying:

“These days, there’s so much more information about parenting and so many different ideas allegedly, professional ideas about what makes a good parent. What’s appropriate or not appropriate, or, you know, if you do this, this is the long-term consequence it’s going to have on your child.”

In a similar vein, Donna—a married Canadian mother of three—explains why she feels it is much harder to be a good parent today: “because you’re bombarded with so much information, you know, for every crisis there’s a book about it. And how do you know which one is right?”.

Striking in the participants’ discourses was not only an awareness of the pressures to invest in children, but also the close link that was drawn between that pressure and the notion of personal responsibility. As Annie—a divorced mother of two from America—expresses it: “I felt like he’s my child and I should be able to take care of him. I mean like when you choose to have a child, they’re your responsibility, not the government’s, you know”. Similarly, Shannon—a married mother of two from America—emphasize personal responsibility in stating: “There’s only a few people that will advocate for your children and that’s you. If you don’t advocate for your kids, guess what, you better roll over, because nobody else is going to do it for you”.

The combined pressure on mothers to invest in their children and to take full responsibility for them was therefore not only large; failure to meet the high expectations was perceived very negatively. As Jenna—a divorced Canadian mother of three—puts it: “If the parents don’t care enough to drive their kids around, and take them, and make sure they have what they need, you lose out. Your kids lose out.”

### Motivation: personal growth and satisfaction from doing a good job

The alternative theoretical motive for investing in children is that allocating time and money to children brings intrinsic rewards for the parents themselves, specifically in terms of personal growth. Mothers in our sample not only devoted a lot of time to their children, but ultimately enjoyed doing so, and wished they could spend even more time with them. As Emily—a married mother of two from Canada—phrases it: “I enjoy it. I like being able to spend time with my boys”. Tina—a cohabiting mother of two from America—echoes this sentiment by saying: “It’s very rarely I go out by myself. Just prefer to be with them than anybody else”. Thus, while mothers perceive pressures to devote time to their children, it appears that they also derive satisfaction from doing so. As Ashley—a divorced mother of two from Canada—puts it:

“I’ve been a mother for so many years (…) And this is one job that I have done to the absolute best of my ability for almost 20 years and I’ve done a damn good job of it (…) I sometimes think that if I could take that commitment and dedication to the job that I have as a mom, and put it to anything else I’d be hugely successful.”

Similarly on reflecting on her experience as parent, Carol—a married mother of two from Canada—reaches the conclusion towards the end of the interview that: “I think that my husband and I are both excellent parents, and I think it shows in the way our kids are turning out [laughs].”

But if participants in our sample derived satisfaction from doing a good job as parent, few explicitly phrased it in terms of personal growth. Annie—a divorced American mother of two—is a bit of an exception in saying:

“But he’s also brought out the best in me and made me realize a lot more who I am, you know. Does that make sense? So thank God for him because he’s made me a better person, you know, brought out more patience than I ever thought I had in my life. [laughter]”.

Such a perception fits the notion of having children as providing an opportunity for self-growth.

At the same time, participants did acknowledge that this investment in their children came at the expense of their own time and involved much self-sacrifice. To the question: “Do you feel you’re in control of your own time?”, Mary—a divorced mother of three, also from America—replies: “My own time? No, I’m not in control of my own time at all. I’m at the mercy of everyone else. My own time might be, uhh, very late at night. Between 10 and 1.” Jenna—a divorced mother of three from Canada—goes in the same direction stating: “It’s- it’s kind of- uhh, my life is on hold. Umm, I don’t have much of a social life”.

What was however particularly interesting is that mothers viewed this investment as a finite one on their time horizon, that is, an investment that would decrease or even disappear when the children would be older. For example, Jackie—a married mother of three from America—reasons:

“Well, I don’t have much time to myself but that’s not gonna change right now, I mean until they’re a little older, you know, when they’re a little bit more dependent of themselves and make them do things. You know, right now it’s mommy for everything.”

When being asked at the end of the interview how she sees her life developing in the next 5 years, Mia—a divorced Canadian mother of two—says: “Next five years? I hope my daughter will go to the university, and my son will be in high school, so I think my life will be easy”. Jenna—a divorced mother of three, also from Canada—says: “I’m hoping that I’ll be able to have a little bit of life for myself”.

In other words, and while the child-centered parenting practices adopted by parents do bring some rewards in terms of enjoying being with the children and in deriving satisfaction from doing a good job, it is also clear that they involved large self-sacrifice. And yet, this self-sacrifice is perceived as inevitable in order to be a good parent.

## Discussion

We started this paper by presenting three theoretical perspectives that could help explain the current high level of parental investment into children as currently observed in low fertility countries. Our aim was to assess the extent to which these three perspectives figured in parents’ discourses, and whether they were used by parents to justify their investment into children.

With regard to the first perspective, we did find evidence of the importance parents attached to education and future labour market prospects to motivate their investment into their children. Mothers held high educational aspirations for their children, attempted to choose the best schools for them, intervened on their behalf when they were dissatisfied with the school or teachers, and found additional resources when their children were having learning difficulties. This is in line with the argument that investing in children’s human capital is perceived as the best way of preparing them for the labour market and is a prime reason for parental investment (Doepke, Sorrenti, & Zilibotti, 2019; Duncan & Murnane, 2011).

At the same time, mothers viewed their investment into their children as pursuing much broader goals beyond their strict earnings potential. In particular, non-economic markers of success were widely used by participants in our study when they emphasized the importance of their children being happy, finding a job they love, and fulfilling their dreams. At times, these non-economic markers of success even placed education in a secondary position. This finding does not fit with a strict economic perspective. Instead, it could indicate that a broader set of skills, beyond an academic diploma or degree, is needed today to succeed in life. If so, this would be in line with the concept of concerted cultivation as suggested by Lareau (2002), which involves helping children acquire education, but also social and cultural capital. Nevertheless, it could also be that this finding points to deep cultural and cross-national differences in the importance attached to education. We come back to this issue below.

The second theoretical perspective that we examined stressed the importance of social norms as determinant of parental investment into children. In particular, the literature argued that the emergence of new parenting ideology, and norms surrounding what makes a good parent and a good citizen, places high pressures on parents to devote a high level of resources to their children. Our data provided support for this theoretical perspective. For example, mothers referred to the high social pressures that they perceived to devote much time and energy to their children, to be there for them, and to adopt a very child-centered parenting style. This is fully in line with the concept of intensive mothering suggested by Hays (1996) and the child-rearing logic of concerted cultivation suggested by Lareau (2003).

Mothers in our sample however also felt torn between conforming to these societal expectations on the one hand, and a personal desire for less hectic schedules on the other. There was also a perceived pressure to rely on experts to maximize child development opportunities, while feeling overwhelmed by the amount of information available and the large amount of time that it entails to read on these advices and to act on them. In turn, this resulted at times in anxiety in terms of questioning whether they were doing it right as parents. The pressure to conform to the norms of intensive parenting, the reluctance to do so, as well as the perceived risks of not doing so was also reported in recent studies (Smyth & Craig, 2016).

The third theoretical perspective considers the experience of parenting, including the high level of parental investment, as part of the overall trend towards individualization and as contributing towards the parents’ personal growth and self-development. We found some support for this, especially in the rewards and satisfaction of doing a good job as parent, which implicitly requires large time and financial investment into children. This confirms recent findings that despite the high demands associated with intensive parenting, parents derive satisfaction from endorsing such standards (Le & Impett, 2019). However, it was also interesting to see that mothers saw this as a finite investment, that is, as one that would last while the children were young. Thus, while having children is indeed not incompatible with post-modernist values, as argued by van de Kaa (1998), the sacrifices that were involved in being a good parent led participants to put their own needs and self-growth temporarily aside while they were devoting themselves fully to their children.

Crucially, while the literature tends to discuss these different motives separately, the findings clearly suggest an interconnection between them. Todays’ parents wish their children to do well, want what is best for them, and feel responsible to provide it. The highest goal they seem to have in mind for their children is being happy and self-fulfilled. To achieve this nowadays, education and human capital are perceived as being important, perhaps not as much as an end in itself, but as a means to realize this higher goal. Additionally, helping their children to reach their full potential appears a way for parents to enhance their feelings of competence, and thus contributes to their own self-fulfillment. This leads to a more complex interpretation of the motives for parental investment into children, but also one that more closely mirrors the data. It further suggests the formidable challenges and juggles associated with allocating one’s time and financial resources to children, one which takes into account future return (good and successful children), current consideration (self-sacrifices), and societal pressures.

### Specificities of the context

Our qualitative data provided us with a unique vantage point to probe into parents’ perception and experiences of their allocation of time and money to their children and their related motives. However, as our data pertained to a very specific group of middle-income parents and a very specific national context, it remains unclear to what extent our results are generalizable beyond this specific group. Nevertheless, there are some indications that the results have broader validity.

First, the heterogeneity of our sample in terms of its social background, education level, and occupation suggest that the economic motives and social pressures to invest in children are not unique to a very small segment of the population, but are likely to be shared more widely. This is in line with other studies that have suggested that the concept of intensive parenting is also shared more widely in the population (Gauthier et al., 2020; Nomaguchi & Milkie, 2020). People from different income groups have unequal means to act on the norms of intensive parenting, but it is likely that they have the same economic, normative, and psychological motivation to invest in children. However, this is something that should be tested empirically on larger population samples, especially since it would contradict the thesis of Lareau regarding social class differences in childrearing ideology.

Second, and while the sample was confined to Canada and the USA, it can be argued that global changes in terms of income inequality, precarious labour market, and individualism are producing conditions to invest in children that apply equally for a large number of countries (Rao, 2018; Schneider, LaBriola, & Hastings, 2018). This argument is supported by the fact that, although the ideology of intensive mothering is one that has been most explored in Anglo-Saxon countries, it is also starting to emerge as an important theme in other countries (Faircloth et al., 2013). If so, it would suggest that it is not an ideology specific to North America or the countries belonging to a liberal welfare state regime, but that it has a more global relevance. What is however lacking here are cross-nationally comparative studies to examine this. The recent study by Gauthier et al. (2020) is a first step in this direction and suggests indeed the relevance of intensive parenting to a broader set of countries.

Where country specificities may however be important is our findings concerning the importance of non-economic markers of success as a motivation for parental investment. There is a solid literature pointing to deep cultural differences when it comes to the centrality of education. For example, Hess, Chang, and McDevitt (1987) have shown how Asian parents place a larger importance on education and hard work as a way to succeed while American parents emphasized achievement in school to a lower extent. Similarly, the literature on ‘Tiger Moms’ has highlighted the very different approach to parenting held by American Asian mothers (Chua, 2011). The participants in our sample did stress the importance of education, but also acknowledged other markers of success. Consequently, we may expect larger cross-national differences in the degree to which the perceived importance of education is a driver for parental investment. This is most likely the case in East Asia, where the highly competitive education system motivates parents to invest heavily in their children (Anderson & Kohler, 2013).

## Conclusion

This paper aimed at gaining a better understanding of the motives for the high parental investment in children that has been observed over the past decades. Specifically, we aimed at assessing the extent to which elements drawn from three different theoretical perspectives were prominent in mothers’ own account of their parenting practices.

Our empirical findings supported each of these three perspectives. They highlighted (i) the perceived importance attached to economic—but also non-economic—markers of success, (ii) the strong social pressures on mothers to adhere to an intensive parenting ideology, and (iii) the personal reward and feeling of self-fulfillment derived from investing into children. They also pointed to important interconnections between the three perspectives involving both personal and societal considerations, both parents’ and children’s own growth and self-fulfillment, and an important time horizon by which current sacrifices are traded off for later rewards.

In addition to bringing new insights into contemporary discourses of parenting and their determinants, our study may also be relevant for our theoretical understanding of fertility. In particular, what the study reveals is that it is not only the prospect of ‘quality’ children that motivates today’s parents to invest in their children, but also the aim and experience associated with bringing up happy, well connected, and articulated children. Thus, while today’s norms of good parenting put pressures on parents to devote considerable amounts of time and financial resources to provide their children with a good head start in life, parents also derive a sense of reward from their large investment.

In this paper, we did not compare the motivations for parental investment of families with different numbers of children, and therefore cannot speak directly about the link with fertility decisions. However, what the findings do suggest is that the large personal and societal expectations of good parenting put considerable pressures on parents to invest in their children. In turn, these investments have an undeniable impact on the actual cost of children, and may be only compatible with a small number of children (Lebano & Jamieson, 2020; Ogawa, Mason, Chawla, Matsukura, & Tung, 2009). This not only opens up interesting avenues of research, but also calls for the development of quantitative instruments to capture contemporary norms of good parenting, parental investment, and related motivations which go beyond what is routinely done in large scale surveys. Such data, and such a broader conceptualization of parental investment, would allow for a better understanding of the role that it plays in fertility decisions in low fertility countries.

## Data Availability

The datasets at the basis of the current study are not publicly available since the original ethics certification (referred to in the manuscript) restricted its use to the original study team members and associates. However, the various memos presenting the results and quotes are available from the corresponding author on reasonable request.

## Abbreviations

STD: Second demographic transition

## Footnotes

1 There have however been increasing calls to expand this economic definition to include other dimensions such as parenting skills, style, and attention.

2 One additional theoretical perspective on parental investment that is not covered in this paper is the evolutionary one. The reader is referred to Kaplan (1996) for this perspective.

3 The term ‘English-speaking’ obviously overlooks the large proportion of French-speaking population in the Canadian province of Quebec.

4 Some studies have also suggested differences between the two countries in the size of the middle class (Pressman, 2007).

5 Detailed information about the study and the research team is available in Gauthier (2015a). The study received ethics approval of all universities where the principal investigators were located at the time.

6 One key limitation of the sample is an under-representation of ethnic minorities.

7 In 2009, this corresponded to 51,000 euro (using the value of Purchasing Power parity for the Netherlands).

8 This is the number of cases in the first round of data collection. In one of the sites (Philadelphia), a second and third rounds were carried out in which a few additional participations were added.

9 We included in this group one participant which income used to be in the middle-income range but which had very recently decreased due to the loss of a job.

10 Specifically, married or cohabiting (77% USA, 72% Canada), some post-secondary education (85% USA, 83% Canada), employed (77% USA, 72% Canada), homeowner (95% USA, 92% Canada), and middle-class (83% USA, 89% Canada).
